# Etymologia: measles

**DOI:** 10.3201/eid1204.ET1204

**Published:** 2006-04

**Keywords:** Etymologia, measles

## [me′zəlz]

Highly contagious disease caused by a virus of the genus *Morbillivirus*, marked by an eruption of distinct, red, circular spots. From the Middle Dutch *masel*, “blemish.” References to the disease date back to at least 700 ad, but the first recorded scientific description of measles was in the 10th century ad by the Persian physician Ibn Razi, who described it as “more dreaded than smallpox.” Prior to 1963, when the first measles vaccine was licensed, 3–4 million cases and 450 deaths occurred in the United States every year. Measles remains a primary cause of death in developing nations, where vitamin A deficiency is common. According to the World Health Organization, measles is the leading cause of vaccine-preventable death in children; it is responsible for ≈850,000 deaths each year.

Sources: Merriam-Webster’s collegiate dictionary. 11th ed. Springfield (MA): Merriam-Webster Incorporated; 2003; Centers for Disease Control and Prevention. Measles history. Available from http://www.cdc.gov/nip/diseases/measles/history.htm; and World Health Organization. Measles mortality reduction and regional elimination. Available from http://www.who.int/vaccines-documents/DocsPDF01/www573.pdf.

